# Postoperative results of minimally invasive direct coronary artery bypass procedure in 234 patients

**DOI:** 10.3389/fcvm.2022.1051105

**Published:** 2023-01-10

**Authors:** Nadejda Monsefi, Eissa Alaj, Sami Sirat, Farhad Bakhtiary

**Affiliations:** ^1^Department of Cardiac Surgery, University Hospital Bonn, Bonn, Germany; ^2^Department of Cardiac Surgery, Heart Center Siegburg, Siegburg, Germany

**Keywords:** minimally invasive, off-pump surgery, minithoracotomy, hybrid procedure, left anterior descending

## Abstract

**Introduction:**

Minimally invasive approach in cardiac surgery has gained popularity. In order to reduce surgical trauma in coronary surgery minimally invasive direct coronary artery bypass (MIDCAB) has already been established. This technique has been introduced for revascularisation of isolated left anterior descending (LAD). It can also be performed for hybrid revascularisation procedure in multi-vessel disease.

**Methods:**

From 2017 to 2021, 234 patients received MIDCAB operation in our heartcenter 73% were male. Most of the patients had two or three vessel disease (74%). The average age of the patients was 66 ± 12 years mean. The left internal mammary artery (LIMA) was anastomosed to the LAD through left minithoracotomy approach. Multi-vessel MIDCAB (MV-MIDCAB) including two anastomoses (T-graft to LIMA with additional saphenous vein graft) was done in 15% (*n* = 35).

**Results:**

The average operation time was 2.3 ± 0.8 h mean. The 30-day mortality was 1.7% (*n* = 4). The average amount of packed red blood cells (pRBC) that was given intra- and postoperatively was 0.4 ± 0.8 units mean. The mean intensive care unit stay (ICU) was 1 ± 1.2 days. Three patients (1.3%) had wound infection postoperatively. The rate of neurologic complications was 0.4% (*n* = 1). Two patients (0.9%) had myocardial infarction and received coronary re-angiography perioperatively including stent implantation of the right coronary artery.

**Discussion:**

The MIDCAB procedure is a safe and less traumatic procedure for selected patients with proximal LAD lesions. It is also an option for hybrid procedure in multi-vessel disease. The ICU stay and application of pRBC’s are low. Our MIDCAB results show a good postoperative clinical outcome. However, follow-up data are necessary to evaluate long-term outcome.

## Introduction

Minimally invasive cardiac surgery for the treatment of valve disease is well-established. Coronary artery bypass grafting (CABG) *via* minimally invasive approach is an outstanding evolution in cardiac surgery. Since the first beating heart anastomosis was described by Kollesov in 1967 (1) the Off-pump bypass surgery technique has been developed continuously during the past decades. Minimally invasive direct coronary artery bypass (MIDCAB) grafting was presented in the 1990s by Calafiore and Subramanian (2, 3). Today it is an important part of the cardiac surgery armamentarium in centers of excellence. MIDCAB procedure is a revascularization strategy for the treatment of left anterior descending (LAD) disease. It can also be applied as a hybrid coronary revascularization (HCR) in the setting of incomplete surgical revascularization for high-risk patients. These patients usually undergo postoperative interventional percutaneous coronary intervention (PCI). In selected patients multi-vessel MIDCAB is feasible to treat lesions of LAD, diagonal branch or circumflex artery. Less surgical trauma, reduced operative bleeding, and fast recovery are associated with MIDCAB approach (4–6). Despite the advantages of MIDCAB procedure this technique has not been widespread in the routine cardiac surgery field. It might be related to the fact that MIDCAB remains technically challenging due to limited access to the surgical situs and limitation of exposure of the heart (5). Another reason could be that CABG and PCI are indexed for class IA category for treatment of isolated proximal LAD lesions in the guidelines on myocardial revascularization (7). Therefore the desicions of heartteams play an important role to enclose the suitable patients for this minimally invasive procedure.

## Patients and methods

### Study population

From 2017 to 2021, 234 patients underwent MIDCAB procedure in Heartcenter Siegburg and University Hospital Bonn. 27% were female. The majority of patients had two or three vessel disease (74%). Patients’ mean age was 66 ± 12 years. The left internal mammary artery (LIMA) was anastomosed to the LAD *via* left minithoracotomy approach in all patients. MV-MIDCAB with two anastomoses (additional saphenous vein graft as T-graft to LIMA) was performed in 35 patients (15%). The patients’ preoperative characteristics are summarized in Table 1.

**TABLE 1:** Patients’ characteristics.

Number, *n*	234
Age (mean, years)	66 ± 12
Male	173 (73%)
NYHA class (mean)	3 ± 0.5
CCS class	3 ± 0.7
One-vessel CAD	62 (26%)
Two-vessel CAD	70 (30%)
Three-vessel CAD	102 (44%)
Ejection fraction (mean, %)	51 ± 10
**Comorbidities**	
Diabetes mellitus type 1	3 (1%)
Diabetes mellitus type 2	45 (19%)
COPD stage 1 (mild)	4 (1.7%)
COPD stage 2 (moderate)	20 (8.6%)
Renal failure stage 2	22 (9.4%)
Renal failure stage 3	23 (10%)
Renal failure stage 4	9 (3.8%)
Renal failure stage 5	1 (0.4%)
Myocardial infarction	70 (30%)
Arterial hypertension (%)	159 (68%)
EUROScore II (mean)	3 ± 3.6
Hemoglobin (mean, gr/dl)	12.6 ± 2
PAD	25 (11%)
PCI, stent	77 (33%)

NYHA, New York heart association; CCS, Canadian cardiovascular society; CAD, coronary artery disease; COPD, chronic obstructive lung disease; COPD stage 1: FEV1 > 80%; COPD stage 2: FEV1 50–80%; PAD, peripheral arterial disease; PCI, percutaneous coronary intervention; diabetes mellitus type 1 (insulin dependent), type 2 (non-insulin dependent); renal failure stadium 2: GFR 60–89 ml/min/1.73 m^2^, renal failure stadium 3: GFR 30–59 ml/min/1.73 m^2^, renal failure stadium 4: GFR 15–29 ml/min/1.73 m^2^, renal failure stadium 5: GFR < 15 ml/min/1.73 m^2^.

This retrospective study was approved by the local ethics committee (#446/21).

### Patient selection criteria

Suitable patients for MIDCAB were discussed in a heartteam for the surgical/hybrid procedure. Inclusion criteria were significant stenosis or occlusion of the proximal or medial LAD for single vessel revascularization. The diagonal branch or ramus intermedius were targets for multi-vessel revascularization. For HCR the right coronary artery and/or circumflex artery were treated with PCI postoperatively. Exclusion criteria were former chest radiation or left thoracotomy (for lung or breast surgery), stenosis of the left subclavian artery, emergency operation, and/or hemodynamically instable patients, or redo CABG.

### Surgical technique

**FIGURE 1:**
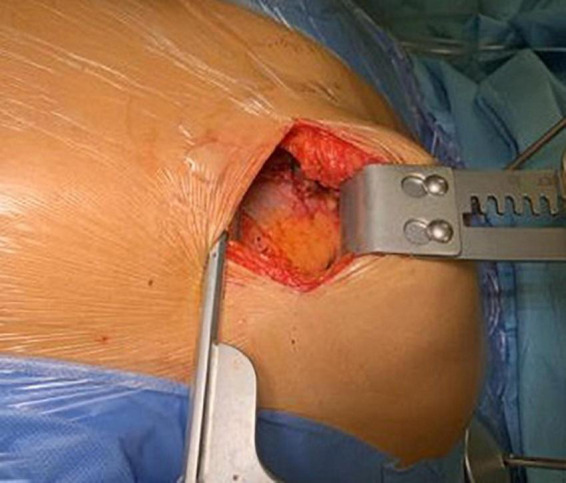
Left thoracotomy for LIMA harvesting using a lifting retractor.

Patients were placed in a supine position, with 30° elevation of the left thorax. Intubation was established with a double-way endotracheal tube. A 5–8 cm long left submammary or supramammary skin incision was done, and the left pleural space was entered through the 4th or 5th intercostal space. The left lung was deflated. With the help of a MICS retractor for LIMA (lifting retractor, Geister, Tuttlingen, Germany) a pedicled LIMA graft was harvested (Figure 1). Systemic heparinization was initiated, the pericardium was opened, and the LAD was identified. The distal anastomosis was performed Off-pump with the help of a vacuum tissue stabilizer (Octopus Evolution, Medtronic, Minneapolis, USA) and an intracoronary shunt (Medtronic, Minneapolis, USA). A traction suture with tourniquet was placed with 4/0 polypropylene to the proximal part of the LAD. Air/saline insufflation was used to achieve a bloodless operation field. Bypass flow was measured routinely intraoperatively. In MV-MIDCAB a small segment of saphenous vein was harvested from the lower leg. The venous graft was anastomosed to the target vessel in a same manner. Finally the proximal anastomosis (T-Graft to LIMA) was performed. If necessary a heart positioner was applied for better exposition (Starfish Evo, Medtronic, Minneapolis, USA). Protamin was administered 1:1, a thorax drain was placed into the left pleura and thoracotomy was closed.

### Statistical analysis

Statistical analyses were calculated with the biometrically analysis of sampling software (BIAS 11.06, Frankfurt, Germany). Categorical data were presented as percentages and continuous data were illustrated as mean value ± standard deviation.

## Results

**FIGURE 2:**
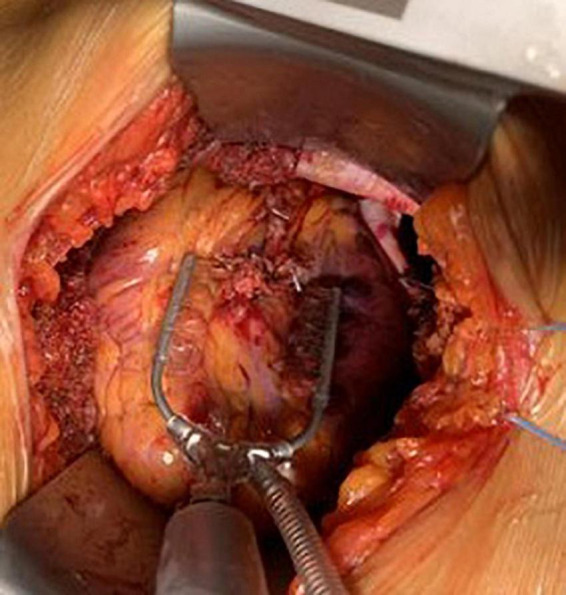
Minimally invasive direct coronary artery bypass, LIMA, to LAD anastomosis.

**FIGURE 3:**
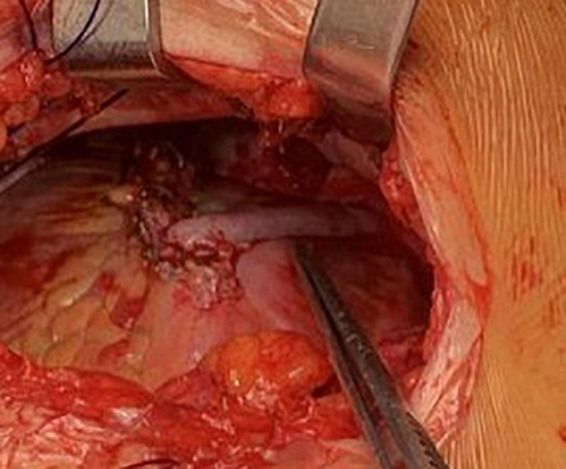
Multivessel-MIDCAB, LIMA, to LAD and saphenous vein (T-graft) to diagonal branch anastomoses.

The majority of patients (85%) received single bypass LIMA to LAD in MIDCAB technique (Figure 2). A total of 15% underwent MV-MIDCAB with LIMA to LAD and saphenous vein (T-Graft) to the diagonal branch (Figure 3). The operative and perioperative results are illustrated in Table 2. The mean operation time was 2.3 ± 0.8 h. Conversion to sternotomy was necessary in one patient (0.4%) who had myocardial ischemia postoperatively. The RCA could not be treated with PCI. Therefore the patient underwent sternotomy for additional bypass to the right coronary artery (RCA) at the first postoperative day. The applied amount of packed red blood cells (pRBC) were 0.4 ± 0.8 units. The average intensive care unit stay (ICU) were 1 ± 1.2 days. One patient (0.4%) presented with a minor stroke postoperatively. Myocardial infarction was observed in two patients (0.9%) who underwent coronary re-angiography perioperatively and stent intervention of the right coronary artery. There was no operative death. The 30-day mortality was 1.7% (*n* = 4). Cause of death were multi organ failure (*n* = 1), low output syndrome (*n* = 2), and sepsis due to pneumonia (*n* = 1). Rethoracotomy for bleeding (*via* left thoracotomy approach) was necessary in eight patients (3.4%). Wound revision due to superficial wound infection was required in three patients (1.3%).

**TABLE 2:** Operative and postoperative results.

**Operative**	
One coronary anastomosis	199 (85%)
Two coronary anastomoses	35 (15%)
Operative time (mean, hours)	2.3 ± 0.8
conversion to sternotomy	1 (0.4%)
Total number of coronary anastomoses	269
**Postoperative**	
Intensive care unit duration (mean, days)	1 ± 1.2
Ventilation time (mean, hours)	6 ± 4
Rethoracotomy	8 (3.4%)
Neurologic event (stroke)	1 (0.4%)
Wound infection	3 (1.3%)
30-day-mortality	4 (1.7%)
Myocardial infarction	2 (0.9%)
pRBC’s (mean)	0.4 ± 0.8
Hemoglobin (mean, gr/dl)	11.3 ± 1
Chest tube output in 48 h (mean, ml)	750 ± 300
Hospital length of stay (mean, days)	6 ± 2

pRBC, packed red blood cells.

## Discussion

Minimally invasive direct coronary artery bypass procedure offers a good solution for patients with isolated proximal LAD stenosis. The avoidance of sternotomy and cardiopulmonary bypass (CPB) in the MIDCAB setting has been associated with faster recovery, less bleeding, and fewer transfusions (8). An important strategy for MIDCAB revascularization is a careful patient selection that should be discussed in a heartteam. A LAD diameter < 1.5 mm, diffuse disease, or intramural position of the LAD are reported to be exclusion criteria for MIDCAB (9). Also unfavorable anatomical conditions like obesity, former chest radiation, left thoracotomy (for lung or breast surgery), or stenosis of the left subclavian artery make MIDCAB unsuitable for these patients. Emergent cases and/or hemodynamically instable patients should be also excluded. In the early beginning of the MIDCAB era this technique was predominantly applied in patients with isolated lesions of the LAD. Nowadays it is an attractive option for HCR in multi vessel disease particularly in high-risk patients with several comorbidities. MIDCAB for HCR is reported to be associated with a favorable clinical outcome including lower major adverse cardiovascular and cerebral events (MACCE) and repeat revascularization rates compared with multivessel PCI (10). In our series 40% of the patients underwent HCR. The 30-day mortality was 1.7% (*n* = 4) that is comparable to the published data of other centers (5, 10–12). Conversion to sternotomy (without cardiopulmonary bypass) was necessary in one patient (0.4%) that is acceptable and similar to published data (5, 10). We observed one (0.4%) neurological event (minor stroke) postoperatively that is low. The applied amount of pRBC were 0.4 ± 0.8 units. The average ICU stay was 1 ± 1.2 days that is short. These findings are similar to the reported results of other MIDCAB performing centers regarding less required blood transfusions and a short ICU stay (5, 13). Although MIDCAB is a challenging technique, it can be performed safely with low complication rates by experienced Off-pump coronary artery bypass (OPCAB) surgeons (11, 13, 14). In selected patients MIDCAB procedure is a good revascularization strategy as described in the following studies. Indja et al. reported that MIDCAB for LAD remains superior to first- or second-generation PCI with DES regarding long-term freedom from myocardial infarction and survival (15). Aziz et al. presented a meta-analysis including 12 studies (1,952 patients) comparing MIDCAB with PCI for single vessel LAD revascularization (16). They could show that there was a higher rate of recurrent angina, need for repeat revascularization and incidence of MACCE with PCI at midterm follow up. Blazek et al. reported the 10-year follow-up results of a randomized trial comparing MIDCAB with bare-metal stenting for stenosis of the LAD (17). They found out that there were no significant differences in the endpoints death and myocardial infarction. However, a significant higher repeat target vessel revascularisation rate was observed in the PCI group (34 vs. 11%, *p* < 0.01). Similar results are described in the propensity matched study of Hannan et al. (18). They observed a significantly lower repeat revascularization rate in patients undergoing CABG vs. PCI with DES (7.09 vs. 12.98%, *p* = 0.0007) in isolated proximal LAD disease at 3-years follow-up. The decision of heartteams plays an important role to enclose the suitable patients for this minimally invasive procedure. There are only a few studies dealing with benefits and late outcomes of heartteam decisions regarding patients with CAD. Domingues et al. report about their experience regarding heartteam recommendations for 1,000 patients with CAD (19). They observed a 5-year mortality rate of 3% for patients with 1 vessel disease with or without proximal LAD involvement. Despite the heartteam recommendation was largely in accordance with the clinical guidelines the timing for treatment could have been further optimized (19). It is mandatory to set up a multidisciplinary heartteam to determine criteria moving a patient for MIDCAB approach. The surgical view regarding the feasibility of minimally invasive approach with/without hybrid strategy in CAD is essential. Therefore the role of cardiac surgeons in heartteam meetings is crucial. The advantage of a hybrid procedure is the revascularization of multiple territories without a large surgical trauma. To set an example, the RCA territory can be treated with PCI afterward MIDCAB LIMA to LAD has been performed. In the most cases it is not possible to reach the RCA *via* left minithoracotomy in off-pump technique. The rate of hybrid procedures in CAD is increasing. Van den Eynde et al. published the results of a systematic review and meta-analysis regarding HCR versus PCI in 27041 patients (20). They observed that HCR was associated with significantly lower rates of myocardial infarction and target vessel revascularization in comparison to PCI. Therefore HCR strategy is gaining popularity in many experienced heart centers as it is a valid alternative to multivessel PCI.

Minimally invasive cardiac surgery can compete with interventional cardiology and offers outstanding results. Although MIDCAB is technically demanding our postoperative results demonstrate that this procedure is safe and feasible (21). Optimal patient selection and an experienced surgical team are mandatory.

Minimally invasive direct coronary artery bypass for selected patients with proximal LAD lesions and in multi-vessel disease is a safe procedure with a low 30-day mortality and good clinical outcome. Intra- and perioperative application of pRBC’s and ICU stay are low. The trauma and incision is small with a good cosmetic result. However, long-term clinical follow up data are necessary to strengthen our thesis.

## Limitations

The study has a retrospective design. A control group, e.g., On-pump CABG, was not added. Follow-up data are not included yet as further investigations are ongoing.

## Data availability statement

The original contributions presented in this study are included in the article/supplementary material, further inquiries can be directed to the corresponding author.

## Ethics statement

The studies involving human participants were reviewed and approved by the University Hospital Bonn. Written informed consent for participation was not required for this study in accordance with the national legislation and the institutional requirements.

## Author contributions

NM and EA analyzed the data. NM, EA, and SS structured the manuscript giving contribute to tables, figures, and text editing. FB revisited the article implementing the final manuscript form. All authors contributed to the manuscript production and in the final revision.
